# A prospective cohort study of postpartum glucose metabolic disorders in early versus standard diagnosed gestational diabetes mellitus

**DOI:** 10.1038/s41598-021-89679-2

**Published:** 2021-05-17

**Authors:** Valeria Cosma, Jeanne Imbernon, Léonore Zagdoun, Pierre Boulot, Eric Renard, Cécile Brunet, Pierre Mares, Michel Rodier, Sarah Kabani, Christophe Demattei, Anne-Marie Guedj

**Affiliations:** 1 Department of Endocrine and Metabolic Diseases, CHU Nimes, Univ Montpellier, Place du Professeur Debré, 30029 Nîmes Cedex 09, France; 2 Service de Médecine, CH Joseph Imbert, Arles, France; 3 Department of Gynecology and Obstetrics, CHU Montpellier, Univ Montpellier, Montpellier, France; 4 Department of Endocrinology, Diabetes, Nutrition, CHU Montpellier, Univ Montpellier, Montpellier, France; 5 Department of Gynecology and Obstetrics, CHU Nimes, Univ Montpellier, Nîmes, France; 6 Department of Biostatistics, Epidemiology, Public Health and Innovation in Methodology (BESPIM), CHU Nimes, Univ Montpellier, Nîmes, France

**Keywords:** Endocrinology, Medical research

## Abstract

Early gestational diabetes mellitus (eGDM) is diagnosed when fasting plasma glucose before 24 weeks of gestation (WG) is ≥ 5.1 mmol/L, whilst standard GDM is diagnosed between 24 and 28 WG by oral glucose tolerance test (OGTT). eGDM seems to have worse obstetric outcomes than standard GDM. We compared the rates of postpartum glucose metabolism disorders between women with early versus standard GDM in this prospective study on women with GDM from three university hospitals between 2014 and 2016. Patients were included if they were < 24 WG with at least one risk factor for GDM and excluded if they had type 2 diabetes. Patients were assigned to Group 1 (G1) for eGDM according to IADPSG: fasting blood glucose < 24 WG between 5.1 and 7 mmol/L. Group 2 (G2) consisted of patients presenting a standard GDM at 24–28 WG on OGTT results according to IADPSG: T0 ≥ 5.1 mmol/L or T60 ≥ 10.0 mmol g/L or T120 ≥ 8.5 mmol/L. The primary outcome was postpartum OGTT result. Five hundred patients were analysed, with 273 patients undergoing OGTT at 4–18 weeks postpartum: 192 patients in G1 (early) and 81 in G2 (standard). Patients in G1 experienced more insulin therapy during pregnancy than G2 (52.2% versus 32.5%, p < 0.001), but no patients were taking insulin postpartum in either group. G1 patients experienced less preterm labour (2.6% versus 9.1%, p = 0.043), more induced deliveries (38% versus 25%, p = 0.049) and reduced foetal complications (29.2% versus 42.0%, p = 0.048). There was no significant difference in the rate of postpartum glucose metabolism disorders (type 2 diabetes, impaired glucose tolerance, impaired fasting glycaemia) between groups: 48/192 (25%) in G1 and 17/81 (21%) in G2, p = 0.58. Thus the frequency of early postpartum glucose metabolism disorders is high, without difference between eGDM and standard GDM. This supports measurement of fasting plasma glucose before 24 WG and the threshold of 5.1 mmol/L seems appropriate until verification in future studies.

**Trial registration**: NCT01839448, ClinicalTrials.gov on 22/04/2013.

## Introduction

In line with the diagnostic criteria of The International Association of Diabetes in Pregnancy Study Group (IADPSG)^1^ and the French recommendations^2^, targeted screening of gestational diabetes mellitus has been recommended for all high-risk women since 2010 in France, with a fasting plasma glucose test before 24 WG. The aim of this screening is to diagnose pre-existing diabetes (> 7 mmol/L), but if the fasting glycaemia is between 5.1 mmol/L and 7 mmol/L, a diagnosis of early GDM (eGDM) is made. If the fasting glucose is < 5.1 mmol/L, an oral glucose tolerance test (OGTT) is performed between 24 and 28 WG and the diagnosis of standard GDM is made if at least one value is abnormal (T0 ≥ 5.1 mmol/L, T60 ≥ 10.0 mmol/L and T120 ≥ 8.5 mmol/L).

eGDM emerges in a dysmetabolic environment and could represent an intermediary between pre-existing diabetes and standard GDM^3^. It is characterised by a high and early insulin resistance from the first trimester of pregnancy, but can also arise from defective beta-cell function as interpreted by abnormal insulin sensitivity tests^4^. The risk factors are similar to those of type 2 diabetes: advanced age, family history of diabetes, personal history of GDM or previous macrosomia^3^. However, its medical care is controversial and the fasting plasma glucose threshold of 5.1 mmol/L has been decided arbitrarily. Indeed, it comes from the HAPO study^5^ and corresponds to the threshold of a fasting plasma glucose between 24 and 32 WG associated with an increased obstetrical risk of 1.75 times compared to a normal pregnancy. The cut-off of 5.1 mmol/L has been generalised to the first trimester for simplicity, but has not been evaluated in prospective studies. In addition, the HAPO study shows a continuum between the fasting plasma glucose level and the incidence of obstetrical complications. This argues against applying a single threshold^5^. The literature shows contradictory results on the persistence of eGDM, with some studies demonstrating that less than 50% of eGDM persists between 24 and 28 WG^6,7^, whereas one pilot study found that 89% of women with eGDM also returned positive results at 24–28 WG^8^. Interestingly, this study showed a potential benefit from early diagnosis and prompt treatment on reducing the cases of large for gestational age babies, but offset by an increase in babies hospitalised in neonatal intensive care. Several studies have shown no maternofoetal benefit from treated early GDM versus treated standard GDM, especially regarding foetal macrosomia^9^ (7). Indeed, overtreatment of eGDM could be dangerous for the foetus^10^, and the risk/benefit ratio of eGDM screening has not been studied. The effects of eGDM are unknown, especially regarding obstetric outcomes and metabolic future of the mother and the baby.

Thus our study aims to deepen the knowledge of the metabolic impact of eGDM in the early postpartum state in mothers. Our objective is to compare the rate of early postpartum glucose metabolism disorders after eGDM and standard GDM.

## Methods

This prospective and multicentric study was conducted in the Gynaecology and Endocrinology Departments at the University Hospital of Nîmes, the University Hospital of Montpellier and Regional Hospital of Arles, France from March 2014 until October 2016. The study was approved by the research ethics committee CPP Sud Mediterranee III and was performed in accordance with the declaration of Helsinki; it was registered on clinicaltrials.gov (NCT01839448) on 22/04/2013. Participants were pregnant women of less than 24 WG who had at least one risk factor for gestational diabetes: age > 35 years old; BMI > 25 kg/m^2^; family history of type 2 diabetes; personal history of GDM; or a previous macrosomic baby. Eligible patients had to meet criteria of gestational diabetes diagnosis according to the IADPSG, be aged > 18, be available for a 10 month follow-up and give informed consent. They were excluded if they had a pre-existing type 2 diabetes (known or discovered during the pregnancy), had a contraindication or a medical interaction with one of the treatments of the study, were treated with corticosteroids or beta-mimetic drugs in the week before the blood test (fasting plasma glucose or OGTT), or could not read French fluently.

Participants were seen by their midwives or gynaecologists before 24 WG. If they had at least one risk factor of GDM, they were informed about the study, the importance of the postpartum OGTT and their written consent was collected. They were then prescribed a blood test with fasting plasma glucose, to be done at their local laboratory before 24 WG. Group one (G1) consisted of patients presenting an eGDM according to IADPSG: fasting blood glucose on venous samples before 24 WG between 5.1 mmol/L and 7 mmol/L inclusive. If the fasting blood glucose before 24 WG was < 5.1 mmol/L, a 75 g OGTT was performed on venous samples between 24 and 28 WG. Group two (G2) consisted of patients presenting a standard GDM at 24–28 WG according to IADPSG: T0 ≥ 5.1 mmol/L or T60 ≥ 10.0 mmol g/L or T120 ≥ 8.5 mmol/L.

The medical care was identical in the two groups during pregnancy, starting with a consultation in the endocrinology service. Information about GDM and dietary advice based on 200 g carbohydrates intake a day was provided and patients were issued with a capillary blood glucose monitoring system with instructions to perform six tests per day. The patients were seen again after 8 days to check their blood glucose regulation. The glycaemic objectives were fasting plasma glucose < 5.2 mmol/L and 2 h post-prandial plasma glucose < 6.6 mmol/L. For patients with non-controlled blood glucose, insulin therapy was initiated and the patients were seen after 8 days, then followed every 2 weeks. For patients with controlled blood glucose, the capillary blood glucose monitoring was reduced to four controls a day, 3 days a week, and they were seen every month.

After delivery, the patients were contacted by telephone at 4, 6 and 8 weeks postpartum to remind them to do the 75 g OGTT before 12 weeks postpartum at their local laboratory. A final visit with the diabetologist was made after the OGTT to interpret the results and to determine follow-up.

### Outcomes

The main outcome was the result of the 75 g OGTT taken at 4–12 weeks postpartum in both early and standard GDM in order to compare the rate of glucose metabolism disorders (type 2 diabetes, impaired glucose tolerance, impaired fasting glycaemia). The secondary endpoints were: the rate of each glucose metabolism disorder; the rate of obstetrical and maternal complications (caesarean section, gestational hypertension, preeclampsia, urinary tract infection, macrosomia, dystocia, transfer to neonatology, respiratory distress, gestational death, infections and preterm delivery); the presence of risk factors of GDM; macrosomia (birth weight > 4000 g), malformations, neonatal asphyxia, respiratory distress, hypoglycaemia, hypocalcaemia, hyperbilirubinemia, infant death, transfer to Neonatal Intensive Care Unit in both early and standard GDM; a final study outcome was to determine a threshold of fasting plasma glucose before 24 WG to predict the occurrence of glucose metabolism disorder in the postpartum period.

### OGTT analysis

The 75 g OGTT was performed on patients in their laboratory of choice using a standardised protocol. The patient should be fasting for 12 h, at rest and avoid smoking before the exam. The patient ingests 75 g of glucose. Blood glucose is measured prior to ingestion (T0), at 60 min (T60) and at 120 min (T120) via venous sampling. The blood is collected in a tube with a coagulation activator (SST).

The glucose metabolism disorders were defined as: type 2 diabetes (defined by a T120 ≥ 11.1 mmol/L); Impaired Glucose Tolerance (defined by 7.8 ≤ T120 < 11.1 mmol/L); Impaired Fasting Glycaemia (defined by 5.5 ≤ T0  < 7 mmol/L according to the American Diabetes Association^11^). Gestational hypertension was diagnosed when systolic and/or diastolic arterial blood pressure ≥ 140/90 mmHg after 20 WG and preeclampsia defined by gestational hypertension combined with proteinuria ≥ 0.3 g/24 h. Macrosomia was defined as a weight ≥ 4 kg. GDM risk factors recorded were: age > 35 years old; BMI > 25 kg/m^2^; family history of type 2 diabetes; personal history of GDM; or a previous macrosomic baby. Proteinuria was tested in patients with hypertension using Multistix (Siemens), and if positive verified on miction samples with laboratory tests. Weight before pregnancy was self-declared by the patient, and then measured at each follow-up visit. New-born outcomes were taken from the patient file completed by the paediatric team.

### Statistical analyses

The sample size was calculated based on previous studies defining GDM using a fasting glycaemia cut-off of 6.1 or 6.3 mmol/L^12–14^. The anticipated repartition was one third of patients in G1 and two thirds in G2^2^, with an expected 55% postpartum glucose metabolism disorder in G1 and 35% in G2^2,13,14^. To reach an alpha risk of 5% and a statistical power of 90%, 261 patients were needed (87 in G1 and 174 in G2). Due to the high risk of missing data, it was decided to include 500 patients in total (167 in G1 and 333 in G2).

The statistical analyses were conducted by BESPIM of University Hospital of Nîmes and all analyses were carried out using R 3.5.1 (R Development Core Team 2018. R Foundation for statistical computing, Vienne, Austria). Categorical variables were compared using Chi2 or Fisher’s exact test when appropriate. The quantitative variables were compared using Student’s t test for Gaussian continuous variables and Wilcoxon–Mann–Whitney’s test for non-Gaussian continuous variables. Analysis of the primary outcome was adjusted on factors that were not comparable between the two groups in a multivariate logistic regression. Fasting glycaemia before 24 WG was analysed by receiver operating characteristic (ROC) curve in both groups to determine the optimal predictive threshold for postpartum glucose metabolic disorders.

## Results

**Figure 1:**
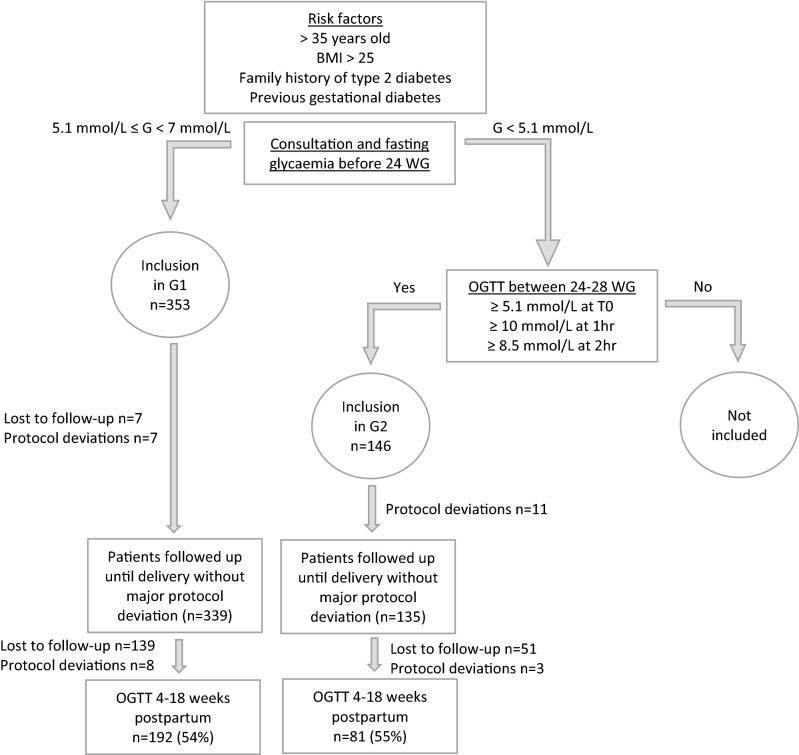
Flow chart. OGTT: oral glucose tolerance test, G: glycaemia.

Five hundred patients were included in the study: 317 at the University Hospital of Nîmes, 168 at Regional Hospital of Arles and 15 at University Hospital of Montpellier. Patients screened due to risk factors for GDM, but not fulfilling the criteria for inclusion into G1 or G2 (no GDM diagnosis at either time point) were not included in the study and thus the number of these patients and their characteristics was not noted. One patient was excluded due to lack of a signed consent form, leaving 353 in G1 and 146 in G2. For the analysis of the primary objective, data from 54% of G1 participants (n = 192) and 55% of G2 (n = 81) were usable (Fig. 1). Patients in G1 underwent the fasting glycaemia at a median of 9.3 [5.7;11.7] WG, and G2 patients took the OGTT at a median of 24.3 [22.7;25.6] WG. The characteristics and medical history of both groups were similar (Table 1) except for initial BMI, which was significantly higher in women in G1 (29.42 ± 5.55 kg/m^2^ versus 26.53 ± 4.45 kg/m^2^, p < 0.001).

**Table 1:** Patient characteristics.

Characteristics	Group 1 (n = 353)	Group 2 (n = 146)	p
**Age**			
Total population (n = 499)	32.82 ± 5.27	32.48 ± 5.29	0.52
Non-analysed patients (n = 226)	31.99 ± 5.45	31.91 ± 5.66	0.92
Analysed patients (n = 273)	33.51 ± 5.03*	32.94 ± 4.96	0.39
**Weight at the beginning of pregnancy (kg)**			
Total population (n = 499)	78.7 ± 16.12	70.27 ± 13.37	** < 0.001**
Non-analysed patients (n = 226)	78.98 ± 16.05	69.57 ± 15.11	** < 0.001**
Analysed patients (n = 273)	78.47 ± 16.21	70.83 ± 11.86	** < 0.001**
**Current weight (kg)**			
Total population (n = 499)	82.16 ± 15.59	78.01 ± 13.13	**0.003**
Non-analysed patients (n = 226)	82.91 ± 15.61	78.41 ± 15.06	0.050
Analysed patients (n = 273)	81.55 ± 15.59	77.69 ± 11.37	**0.027**
**Height (m)**			
Total population (n = 499)	1.63 ± 0.06	1.64 ± 0.06	0.48
Non-analysed patients (n = 226)	1.64 ± 0.06	1.64 ± 0.06	0.63
Analysed patients (n = 273)	1.63 ± 0.07	1.64 ± 0.06	0.59
**BMI before pregnancy (kg/m**^**2**^)			
Total population (n = 499)	29.43 ± 5.67	26.19 ± 4.83	** < 0.001**
Non-analysed patients (n = 226)	29.43 ± 5.83	25.76 ± 5.27	** < 0.001**
Analysed patients (n = 273)	29.42 ± 5.55	26.53 ± 4.45	** < 0.001**
**Family history type 2 diabetes**			
Total population (n = 499)	162 (45.9%)	74 (50.7%)	0.38
Non-analysed patients (n = 226)	74 (46.0%)	34 (52.3%)	0.47
Analysed patients (n = 273)	88 (45.8%)	40 (49.4%)	0.69
**Obstetrical history**			
Total population (n = 499)	292 (82.7%)	114 (78.1%)	0.28
Non-analysed patients (n = 226)	134 (83.2%)	49 (75.4%)	0.24
Analysed patients (n = 273)	158 (82.3%)	65 (80.2%)	0.82
**If yes:**			
**Parity**			
Total population (n = 499)	1 [1;2] (0;7)	1 [1;2] (0;6)	0.10
Non-analysed patients (n = 226)	1 [1;2] (0;7)	1 [1;2] (0;6)	0.35
Analysed patients (n = 273)	1 [1;2] (0;4)	1 [1;2] (0;5)	0.18
**Gestations**			
Total population (n = 499)	2 [1;3] (1;7)	2 [1;3] (1;8)	0.81
Non-analysed patients (n = 226)	2 [1;4] (1;7)	2 [1;3] (1;8)	0.36
Analysed patients (n = 273)	2 [1;3] (1;7)	2 [1;3] (1;8)	0.54
**History of caesarean**			
Total population (n = 499)	75 (21.2%)	25 (17.1%)	0.36
Non-analysed patients (n = 226)	35 (21.7%)	12 (18.5%)	0.71
Analysed patients (n = 273)	40 (20.8%)	13 (16.0%)	0.46
**History of foetal macrosomia**			
Total population (n = 499)	65 (18.4%)	19 (13.0%)	0.18
Non-analysed patients (n = 226)	36 (22.4%)*	12 (18.5%)*	0.64
Analysed patients (n = 273)	29 (15.1%)*	7 (8.6%)*	0.21
**History of GDM**			
Total population (n = 499)	87 (24.6%)	24 (16.4%)	0.06
Non-analysed patients (n = 226)	40 (24.8%)	13 (20%)	0.55
Analysed patients (n = 273)	47 (24.5%)	11 (13.6%)	0.06

Statistically significant p values between groups are given in bold.

Data are presented as number (%), average ± standard deviation or median [Q1; Q3](min; max). *p < 0.05 for the comparison between Non-analysed and Analysed patients.

In total, 284 participants underwent a postpartum OGTT, at a medium term of 10.7 weeks for G1 and 10.4 week for G2. However, 31% of tests were performed later than 12 weeks, thus it was decided to extend the limit of testing until 18 weeks. The 10 patients who took the test beyond 18 weeks were not included in the analysis and neither was one patient for whom the 120-min OGTT result was missing. Finally, 273 participants were assessed for the primary outcome: 192 in G1 and 81 in G2. The global rate of glucose metabolism disorders in the early postpartum period did not differ between the two groups: 25% (48/192) in G1 versus 21% (17/81) in G2, p = 0.58 (Table 2). Similarly, there was no difference in the rate of each type of glucose metabolism disorder between groups: no cases of type 2 diabetes in either group; 9.4% (18/192) impaired glucose tolerance in G1 versus 8.6% (7/81) in G2, p = 1; and 18.8% (36/192) impaired fasting glycaemia in G1 versus 14.8% (12/81) in G2, p = 0.54. No patients were under insulin treatment. Details of pre- and postpartum glycaemia are listed in Supplementary Table 1. Analysis of primary outcome was adjusted on BMI and history of GDM in a multivariate logistic regression. The global rate of glucose metabolism disorders in the early postpartum period did not differ between the two groups after adjustment (p = 0.685).

**Table 2:** Abnormal postpartum glucose metabolism.

	Group 1 (n = 192)	Group 2 (n = 81)	p
Abnormal postpartum glucose metabolism	48 (25.0%)	17 (21.0%)	0.58
Type 2 diabetes (OGTT: T120 > 11.1 mmol/L)	0 (0%)	0 (0%)	–
Glucose Intolerance (OGTT: T120 min: ≥ 7.8 mmol/L and < 11.1 /L	18 (9.4%)	7 (8.6%)	1
Abnormal fasting blood glucose (OGTT: T0 min: > 5.5 mmol/L and ≤ 7.7 mmol/L	36 (18.8%)	12 (14.8%)	0.54

Data are presented as number (%).

During pregnancy, the G1 patients experienced more insulin therapy than G2 (52.2% versus 32.5%, p < 0.001), less preterm labour (2.6% versus 9.1%, p = 0.043), less weight gain (6.82 ± 6.22 versus 9.03 ± 5.77 kg, p = 0.006) and more induced deliveries (38% versus 25%, p = 0.049) (Table 3).

**Table 3:** Pregnancy and delivery characteristics and obstetrical outcomes.

Pregnancy and delivery characteristics	Group 1 (n = 353)	Group 2 (n = 146)	p
**Term (WG)**			
Total population (n = 499)	39.09 ± 1.92	39.42 ± 1.4	0.14
Non-analysed patients (n = 226)	39.14 ± 2.08	39.48 ± 1.33	0.35
Analysed patients (n = 273)	39.05 ± 1.78	39.37 ± 1.47	0.26
**Insulin therapy**			
Total population (n = 499)	169 (49.1%)	49 (34.8%)	**0.005**
Non-analysed patients (n = 226)	63 (41.4%)	24 (37.5%)	0.65
Analysed patients (n = 273)	106 (52.2%)*	25 (32.5%)	** < 0.001**
**Preeclampsia**			
Total population (n = 499)	9 (2.6%)	1 (0.7%)	0.29
Non-analysed patients (n = 226)	4 (2.7%)	0	0.32
Analysed patients (n = 273)	5 (2.6%)	1 (1.3%)	0.68
**Thrombocytopenia**			
Total population (n = 499)	10 (2.9%)	4 (2.8%)	1
Non-analysed patients (n = 226)	4 (2.7%)	0	0.32
Analysed patients (n = 273)	6 (3.2%)	4 (5.2%)	0.48
**Gestational hypertension**			
Total population (n = 499)	11 (3.2%)	2 (1.4%)	0.36
Non-analysed patients (n = 226)	5 (3.4%)	1 (1.6%)	0.67
Analysed patients (n = 273)	6 (3.2%)	1 (1.3%)	0.68
**Preterm labour**			
Total population (n = 499)	10 (2.9%)	10 (7.1%)	**0.045**
Non-analysed patients (n = 226)	5 (3.3%)	3 (4.7%)	0.70
Analysed patients (n = 273)	5 (2.6%)	7 (9.1%)	**0.043**
**Urinary tract infection**			
Total population (n = 499)	11 (3.2%)	6 (4.3%)	0.59
Non-analysed patients (n = 226)	5 (3.3%)	4 (6.3%)	0.46
Analysed patients (n = 273)	6 (3.2%)	2 (2.6%)	1
**Weight gain during pregnancy (kg)**			
Total population (n = 499)	7.56 ± 6.33	9.82 ± 5.52	** < 0.001**
Non-analysed patients (n = 226)	8.48 ± 6.38	10.78 ± 5.09	**0.003**
Analysed patients (n = 273)	6.82 ± 6.22*	9.03 ± 5.77*	**0.006**
**Labour induction**			
Total population (n = 499)	127 (36.8%)	43 (29.9%)	0.15
Non-analysed patients (n = 226)	54 (35.3%)	23 (35.9%)	1
Analysed patients (n = 273)	73 (38.0%)	20 (25%)	**0.049**
**Failed labour induction**			
Total population (n = 499)	23 (18.5%)	5 (11.9%)	0.47
Non-analysed patients (n = 226)	16 (29.6%)	3 (13.6%)	0.24
Analysed patients (n = 273)	7 (10%)*	2 (10%)	1
**Dystocia**			
Total population (n = 499)	31 (9.5%)	16 (11.6%)	0.50
Non-analysed patients (n = 226)	15 (10.6%)	7 (11.1%)	1
Analysed patients (n = 273)	16 (8.6%)	9 (12%)	0.49
**Caesarean**			
Total population (n = 499)	109 (31.6%)	23 (16%)	** < 0.001**
Non-analysed patients (n = 226)	56 (36.6%)	9 (14.1%)	**0.001**
Analysed patients (n = 273)	53 (27.6%)	14 (17.5%)	0.09
**Planned caesarean**			
Total population (n = 499)	46 (42.2%)	10 (43.5%)	1
Non-analysed patients (n = 226)	22 (39.3%)	6 (66.7%)	0.16
Analysed patients (n = 273)	24 (45.3%)	4 (28.6%)	0.36
**Obstetrical outcomes**			
Maternal complications			
Total population (n = 499)	63 (18.3%)	21 (14.6%)	0.36
Non-analysed patients (n = 226)	29 (19.1%)	7 (10.9%)	0.17
Analysed patients (n = 273)	34 (17.7%)	14 (17.5%)	1
Macrosomia (birth weight ≥ 4000 g)			
Total population (n = 499)	32 (9.4%)	12 (8.3%)	0.85
Non-analysed patients (n = 226)	18 (11.8%)	6 (9.5%)	0.80
Analysed patients (n = 273)	14 (7.4%)	6 (7.4%)	1
Foetal complications			
Total population (n = 499)	106 (30.7%)	53 (36.6%)	0.24
Non-analysed patients (n = 226)	50 (32.7%)	19 (29.7%)	0.75
Analysed patients (n = 273)	56 (29.2%)	34 (42.0%)	0.048
Birth weight (g)			
Total population (n = 499)	3344.07 ± 555.88	3337.87 ± 454.01	0.41
Non-analysed patients (n = 226)	3415.41 ± 582.2	3338.02 ± 446.56	0.10
Analysed patients (n = 273)	3287 ± 528.52	3337.75 ± 462.55	0.72

Statistically significant p values between groups are given in bold.

Data are presented as number (%) or average ± standard deviation. *p < 0.05 for the comparison between Non-analysed and Analysed patients.

A significant difference was seen for a reduction of foetal complications in the eGDM group: 29.2% in G1 versus 42.0% in G2, p = 0.048. No significant difference for other obstetrical outcomes was found between the two groups (Table 3).

Analysis of the ROC curve did not identify a discriminatory fasting plasma glucose threshold to predict post-partum dysglycaemia: the area under the curve was 0.579, 95% CI = [0.498;0.660].

There were several differences between the ‘analysed’ (n = 273) vs ‘non-analysed’ (n = 226) patients. Compared to the patients analysed, the patients who did not undergo a postpartum OGTT were significantly younger in G1 (31.99 ± 5.45 versus 33.51 ± 5.03 years old, p < 0.05), had more personal history of macrosomia in G1 (22.4% versus 15.1% p < 0.05) and in G2 (18.5% versus 8.6%, p < 0.05), less insulin therapy in G1 (41.4% versus 52.2%. p < 0.05), more weight gain in G1 (8.48 ± 6.38 versus 6.82 ± 6.22 kg. p < 0.05) and in G2 (10.78 ± 5.09 versus 9.03 ± 5.77. p < 0.05) and more failure of labour induction in G1 (29.6% versus 10%. p < 0.05) (Table 1).

## Discussion

Our results show a high rate of glucose metabolism disorders in the early postpartum period, with the same prevalence in eGDM (25%) as in standard GDM (21%). Considering the high prevalence of eGDM in our population and the fact that less than 50% of early GDM persists after 24 WG^6,7^, screening before 24 WG appears to be valuable to avoid missing glucose metabolism disorders in many women at high risk in postpartum. This high rate of GDM in our study, particularly in early pregnancy, is likely at least partially due to the risk factor-based screening based on the French recommendations, which differ from the systematic screening recommended by the IADPSG. Measuring fasting plasma glucose levels before 24 WG using a threshold of 5.1 mmol/l seems justified in terms of screening for maternal glucose disorders in postpartum.

The impact of early diagnosis of GDM on immediate postpartum dysglycaemia (impaired tolerance glucose and diabetes) versus standard GDM has been reported by Sweeting in 2016^15^. The distribution between normal plasma glucose values, impaired glucose tolerance, and diabetes identified by the OGTT performed 3 months postpartum significantly differed according to onset of GDM: GDM < 12 WG (normal glucose tolerance 79%, impaired glucose tolerance 11%, and diabetes 11%); GDM 12–23 WG (71%, 24%, and 5%, respectively); and GDM > 24 WG (85%, 14%, and 1%, respectively). Hence, the proportion of dysglycaemia was 22% in women with GDM < 12WG, but only 15% in GDM > 24 WG. In a study evaluating pathophysiological characteristics of pregnant women diagnosed with GDM according to IADPSG criteria, the women with eGDM had decreased insulin sensitivity which remained significant after adjustment for BMI, age, and history of GDM^16^.

eGDM was particularly prevalent in our study, representing 70.7% of all GDM. The prevalence of eGDM seen here is higher than in the existing literature: eGDM represents 10.3% of total GDM (universal screening method) in the university hospital of Bondy (n = 9795)^9^, 27.4% in an Australian study (n = 4873)^15^, 34% in Qatar (n = 801)^17^, 48.9% (risk factor screening method) in the university hospital of Lille (n = 334)^10^ and 48.8% more recently in Italy (n = 3974)^18^. In addition to the different screening methods used, the difference in prevalence could be explained by confounding risk factors not recorded here (e.g. ethnic origins, exposure to nutritional factors, or local endocrine disruptors)^19^. A higher incidence of GDM diagnosed in early versus standard screening was also seen in a study in another group of patients at high risk of gestational diabetes due to obesity (15% early versus 12.1% standard)^20^. A Japanese study also found a much higher incidence of eGDM in a population of patients diagnosed with GDM (63.6% < 15 WG versus 36.4% between 24 and 28 WG)^21^. The high rate of eGDM found is not likely to be due to incorrect blood glucose measurements. Fasting glycaemia was tested on venous samples in laboratories conforming with standard practice, negating the need for rechecking at the hospital. Recently, Nakanishi et al. found that women with positive OGTT results taken before 20 WG showed normal results by 24–28 WG in 47%^22^, as previously shown^6,7^. However, the discrepancies between the early and later tests were particularly marked for women where only the fasting result was abnormal in the early test.

In our study, no participant was diagnosed with type 2 diabetes in the early postpartum period. Three possibilities are likely; first, many patients did not complete the postpartum OGTT (41% in G1 and 37% in G2). These non-analysed patients had a higher metabolic risk than the ones analysed; they more frequently had a history of macrosomia and had a significantly higher weight gain during pregnancy. Thus it is possible that we missed some patients exhibiting type 2 diabetes in this group. Also, our diagnostic criterion for type 2 diabetes was a glycaemia two hours after oral glucose load ≥ 11.1 mmol/L. The fasting plasma glucose ≥ 7 mmol/L was not considered as a criterion in this study, whereas one patient in each group exceeded this threshold. These patients were referred to their general practitioner for follow-up, but the data on a second fasting plasma glucose value were not collected as part of this study to establish the diagnosis of type 2 diabetes. It is also possible that type 2 diabetes develops after 18 weeks postpartum. In a systematic review including eight studies and 4026 women with GDM defined with numerous criteria, women with eGDM had a two-fold increased risk of incident type 2 diabetes from 6 weeks to 20 years after delivery compared to subjects with “late” GDM (relative risk (RR) 2.13 (95% CI 1.52–3.56))^23^.

There was a reduction of foetal complications in the eGDM group. This could be the result of a better glycaemic control due to more insulin therapy in G1 and a lower gestational weight gain in G1. Previously, a better glycaemic control was observed by Vambergue et al.^24^ in eGDM: average HbA1c was 5.1 ± 0.4% (32 ± 4 mmol/mol) in eGDM versus 5.3 ± 0.5% (34 ± 4 mmol/mol) in standard GDM (p < 0.001) with HbA1c ≥ 6% (42 mmol/mol) in 2.3% of eGDM versus 6.5% of standard GDM (p < 0.05). Nevertheless, a continuum of risk in terms of maternal outcome has been demonstrated, with eGDM linked to worse outcome than standard GDM, and for some neonatal outcomes eGDM showed an equal or increased risk than pre-existing type 2 diabetes^15^. However, the benefit of screening and treatment of eGDM in terms of foetal outcomes cannot be confirmed by our study as the sample size was determined for the primary objective; many more participants would have been necessary to unveil differences in neonate outcomes. This is particularly true for macrosomia (> 4000 g) for which the ratio was identical in both groups (Table 3).

Several characteristics of the eGDM group were significantly different to the standard GDM group, likely due to the methodological differences in including patients into these groups at the different time points. Patients in the eGDM group had higher pre-pregnancy BMI and lower gestational weight gain, yet higher post-pregnancy weight. The early group also showed a higher rate of insulin therapy, a higher rate of labour inductions and Caesarean and less pre-term labour. Whilst not meeting the threshold for significance, there was trend for women with a history of GDM to have eGDM in this study, in line with Harreiter et al. who found an odds ratio of 2.74 for eGDM in this population^4^. These different baseline, pregnancy and maternal characteristics have been highlighted in other studies on early versus later GDM diagnosis, and are highly co-dependent. Initial higher BMI associated with lower gestational weight gain and more Caesarean sections has been described in a Japanese study^25^. The authors suggested that earlier initiation of treatment was beneficial in the early group, reducing the number of large-for-gestational age infants. Early diagnosis has been consistently linked to increased insulin therapy^15,20,26,27^. The higher pre-pregnancy BMI is likely responsible for the worse outcomes in the eGDM group, as this is a risk factor for Caesarean section and hypotensive disorders^25^ and provokes greater insulin requirements, leading to worse dysglycaemia^15^. In the DALI study analysing pregnant women with a minimum BMI of 29 kg/m^2^, women with the highest BMI were more likely to be diagnosed with eGDM^28^. The different characteristics of the eGDM group, whereby increased and/or earlier initiation of insulin therapy can protect against potential poor maternofoetal outcomes, provide further justification for early screening.

The main limit of our study was the “lost to follow up” bias (41% G1 and 35% G2), although this rate remains less than in other studies, where drop-out is frequently > 50%^23^. Participants largely dropped out after delivery, possibly because of a lack of time with their new-born babies, a fear of the side effects of the glucose load, the pain involved in giving a blood sample or fear of having an abnormal result^29^. The inversion between the expected ratio G2/G1 = 2 and the observed ratio G1/G2 = 2.4 provoked a slight decrease of the power: using the initial hypothesis of an expected 55% postpartum glucose metabolism disorder in G1 and 35% in G2, the power decreases from 90 to 87%. This limited decrease is due to the stability of the total number G1 + G2 (273 patients instead of 261 needed).

Our study has several strengths. It is the first study in France assessing glucose metabolism disorders in the early postpartum period, where early and standard GDM are defined according to current guidelines. Due to numerous telephone reminders, the postpartum OGTT was completed for more than half of the patients, which is far higher than the national average (< 20% within the first 3 months postpartum)^30^. This study is a prospective and multicentric trial, which guarantees a high standard of proof, and enough patients to make statistically significant inferences.

Our results show a high frequency of early postpartum glucose disorders, for both early and standard GDM. eGDM accounts for two thirds of our patients, although less than 50% of those cases would be expected to persist after 24 WG. Thus, it appears important to continue to screen those with eGDM to identify any patients with high metabolic risk in the early postpartum period. This supports the measurement of fasting blood glucose before 24 WG in high-risk women and application of the current threshold of 5.1 mmol/L in terms of maternal outcomes. An analysis of women enrolled into the DALI trial found that the IADPSG criteria appeared suitable for use in early pregnancy, despite the lack of validation^4^. This study also highlighted the difference in prevalence found when using fasting glycaemia alone in comparison with the other time points of the 75 g OGTT, with 78.5% patients diagnosed with eGDM based on abnormal fasting glucose levels alone and only 21.5% with abnormal results at 60 and/or 120 min. However, this study is not directly comparable to ours as diagnosis was based on the WHO 2013 criteria.

This study cannot conclude on the benefit of screening GDM before 24 WG in terms of improved obstetrical outcomes as a much larger cohort would have been required. However, a randomised controlled trial has recently shown that a panel of maternal and neonatal outcomes was no different in a group of high-risk women diagnosed through screening at 14–20 WG versus those screened at 24–28 WG^20^. Furthermore, a large prospective trial has shown variable pregnancy outcomes according to degree of insulin resistance in GDM, in which GDM with high insulin resistance confers greater risk of poor pregnancy outcomes^31^. This is similar to the findings of Sweeting et al. who found that pre-term delivery, rates of Caesarean section and hypertension were as bad in women with eGDM as in those with type 2 diabetes^15^. Finally the European DALI trial across nine countries found no significant difference in any of the pregnancy outcomes between those diagnosed with eGDM, standard or later GDM or with normal glucose tolerance^28^. Other on-going prospective trials such as the EDoGDM (NCT02740283)^32^ and the follow-up to the TOBOGM^33^ pilot study will provide further information of the foetal outcomes after eGDM.

In conclusion, the frequency of early postpartum glucose metabolism disorders is high, particularly impaired fasting glycaemia. However, there is no significant difference in frequency of these disorders between eGDM and standard GDM. These results support measurement of fasting plasma glucose before 24 WG in at-risk patients. Further studies are needed to confirm the threshold value for elevated glycameia in early pregnancy and to better characterise the foetal and maternal outcomes.

## Supplementary Information

Supplementary Information.

## Data Availability

Data are available upon reasonable request to the corresponding author.

## References

[1] International Association of Diabetes and Pregnancy Study Groups Consensus Panel (2010). International Association of Diabetes and Pregnancy Study Groups recommendations on the diagnosis and classification of hyperglycemia in pregnancy. Diabetes Care.

[2] Guedjanne A-M (2010). When should screening be performed for gestational diabetes?. Diabetes Metab..

[3] Cosson E, Carbillon L, Valensi P (2017). High fasting plasma glucose during early pregnancy: A review about early gestational diabetes mellitus. J. Diabetes Res..

[4] Harreiter J (2016). IADPSG and WHO 2013 gestational diabetes mellitus criteria identify obese women with marked insulin resistance in early pregnancy: Table 1. Diabetes Care.

[5] The HAPO Study Cooperative Research Group (2008). Hyperglycemia and adverse pregnancy outcomes. N. Engl. J. Med..

[6] Zhu W-W (2013). Evaluation of the value of fasting plasma glucose in the first prenatal visit to diagnose gestational diabetes mellitus in china. Diabetes Care.

[7] Corrado F (2012). Correspondence between first-trimester fasting glycaemia, and oral glucose tolerance test in gestational diabetes diagnosis. Diabetes Metab..

[8] Simmons D (2018). The treatment of booking gestational diabetes mellitus (TOBOGM) pilot randomised controlled trial. BMC Pregnancy Childbirth.

[9] Cosson E (2019). Early screening for gestational diabetes mellitus is not associated with improved pregnancy outcomes: An observational study including 9795 women. Diabetes Metab..

[10] Langer O (1989). Glycemic control in gestational diabetes mellitus-How tight is tight enough: Small for gestational age versus large for gestational age?. Am. J. Obstet. Gynecol..

[11] American Diabetes Association (2018). 2. Classification and diagnosis of diabetes: Standards of medical care in diabetes—2018. Diabetes Care.

[12] Kitzmiller JL, Dang-Kilduff L, Taslimi MM (2007). Gestational diabetes after delivery: Short-term management and long-term risks. Diabetes Care.

[13] Schaefer-Graf UM, Buchanan TA, Xiang AH, Peters RK, Kjos SL (2002). Clinical predictors for a high risk for the development of diabetes mellitus in the early puerperium in women with recent gestational diabetes mellitus. Am. J. Obstet. Gynecol..

[14] Schaefer-Graf UM (2009). How do we reduce the number of cases of missed postpartum diabetes in women with recent gestational diabetes mellitus?. Diabetes Care.

[15] Sweeting AN (2016). Gestational diabetes mellitus in early pregnancy: Evidence for poor pregnancy outcomes despite treatment. Diabetes Care.

[16] Bozkurt L (2015). Pathophysiological characteristics and effects of obesity in women with early and late manifestation of gestational diabetes diagnosed by the international association of diabetes and pregnancy study groups criteria. J. Clin. Endocrinol. Metab..

[17] Bashir M (2019). Pregnancy outcomes of early detected gestational diabetes: A retrospective comparison cohort study. Qatar. BMJ Open.

[18] Capula C (2016). A new predictive tool for the early risk assessment of gestational diabetes mellitus. Prim. Care Diabetes.

[19] Plows JF, Stanley JL, Baker PN, Reynolds CM, Vickers MH (2018). The pathophysiology of gestational diabetes mellitus. Int. J. Mol. Sci..

[20] Harper LM (2020). Early gestational diabetes screening in obese women: A randomized controlled trial. Am. J. Obstet. Gynecol..

[21] Maegawa Y, Sugiyama T, Kusaka H, Mitao M, Toyoda N (2003). Screening tests for gestational diabetes in Japan in the 1st and 2nd trimester of pregnancy. Diabetes Res. Clin. Pract..

[22] Nakanishi S (2020). High probability of false-positive gestational diabetes mellitus diagnosis during early pregnancy. BMJ Open Diabetes Res. Care.

[23] Rayanagoudar G (2016). Quantification of the type 2 diabetes risk in women with gestational diabetes: A systematic review and meta-analysis of 95,750 women. Diabetologia.

[24] Vambergue A (2018). Diabète gestationnel précoce versus diabète gestationnel tardif: Analyse des issues de grossesses dans une cohorte de 2948 patientes. Ann. Endocrinol..

[25] Usami T (2020). Comparison of pregnancy outcomes between women with early-onset and late-onset gestational diabetes in a retrospective multi-institutional study in Japan. J. Diabetes Investig..

[26] Bianchi C (2019). Early vs. standard screening and treatment of gestational diabetes in high-risk women—An attempt to determine relative advantages and disadvantages. Nutr. Metab. Cardiovasc. Dis..

[27] Immanuel J, Simmons D (2017). Screening and treatment for early-onset gestational diabetes mellitus: A systematic review and meta-analysis. Curr. Diab. Rep..

[28] Egan AM (2017). Epidemiology of gestational diabetes mellitus according to IADPSG/WHO 2013 criteria among obese pregnant women in Europe. Diabetologia.

[29] Sanderson H (2018). Improving uptake of postnatal checking of blood glucose in women who had gestational diabetes mellitus in universal healthcare settings: A systematic review. J. Clin. Med..

[30] Goueslard K (2017). Early screening for type 2 diabetes following gestational diabetes mellitus in France: Hardly any impact of the 2010 guidelines. Acta Diabetol..

[31] Benhalima K (2019). Characteristics and pregnancy outcomes across gestational diabetes mellitus subtypes based on insulin resistance. Diabetologia.

[32] Liu B (2016). Early Diagnosis of gestational diabetes mellitus (EDoGDM) study: A protocol for a prospective, longitudinal cohort study. BMJ Open.

[33] Simmons D (2018). Hyperglycaemia in early pregnancy: The Treatment of Booking Gestational diabetes Mellitus (TOBOGM) study. A randomised controlled trial. Med. J. Aust..

