# Supplementary material for: A prospective cohort study of postpartum glucose metabolic disorders in early versus standard diagnosed gestational diabetes mellitus
Source: Sci Rep. 2021 May 17;11:10430. doi: 10.1038/s41598-021-89679-2 (PMC8128886; doi:10.1038/s41598-021-89679-2)
Supplement: Supplementary file 1 — Supplementary Information. [file 41598_2021_89679_MOESM1_ESM.pdf]

Supplementary Information

A prospective cohort study of postpartum glucose metabolic disorders in early versus standard diagnosed gestational diabetes mellitus

Valeria Cosma, Jeanne Imbernon, Léonore Zagdoun, Pierre Boulot, Eric Renard, Cécile Brunet, Pierre Mares, Michel Rodier, Sarah Kabani, Christophe Demattei, and Anne-Marie Guedj

**Blood glucose values (mmol/L)**	**Group 1 (n=353)**	**Group 2 (n=146)**	**p**
**Fasting glycemia < 24 WG**			
Total population (n=499)	5.34 ± 0.28	4.62 ± 0.22	**<0.001**
Non-analyzed patients (n=226)	5.34 ± 0.33	4.62 ± 0.28	**<0.001**
Analyzed patients (n=273)	5.34 ± 0.22	4.68 ± 0.22	**<0.001**
**24-28 WG OGTT T0**			
Total population (n=499)		4.79 ± 0.50	
Non-analyzed patients (n=226)		4.79 ± 0.55	
Analyzed patients (n=273)		4.79 ± 0.50	
**24-28 WG OGTT T60**			
Total population (n=499)		9.57 ± 1.54	
Non-analyzed patients (n=226)		9.30 ±1.71	
Analyzed patients (n=273)		9.79 ± 1.32	
**24-28 WG OGTT T120**			
Total population (n=499)		8.09 ± 1.60	
Non-analyzed patients (n=226)		7.92 ± 1.815	
Analyzed patients (n=273)		8.25 ± 1.43	
**Post-partum OGTT T0**			
Total population (n=499)	5.17 ± 0.44	4.90 ± 0.55	**<0.001**
Non-analyzed patients (n=226)	5.335 ± 0.33	4.68 ± 0.39	**0.002**
Analyzed patients (n=273)	5.17 ± 0.44	4.95 ± 0.55	**<0.001**
**Post-partum OGTT T120**			
Total population (n=499)	5.83 ± 1.43	5.72 ± 1.49	0.39
Non-analyzed patients (n=226)	6.05 ± 1.32	5.72± 1.43	0.60
Analyzed patients (n=273)	5.78 ± 1.43	5.72± 1.49	0.43

Supplementary Table 1: pre- and post-partum OGTT results. Data are presented as average ± standard deviation
